# Prediction of MGMT promotor methylation status in glioblastoma by contrast-enhanced T1-weighted intensity image

**DOI:** 10.1093/noajnl/vdae016

**Published:** 2024-02-01

**Authors:** Takahiro Sanada, Manabu Kinoshita, Takahiro Sasaki, Shota Yamamoto, Seiya Fujikawa, Shusei Fukuyama, Nobuhide Hayashi, Junya Fukai, Yoshiko Okita, Masahiro Nonaka, Takehiro Uda, Hideyuki Arita, Kanji Mori, Kenichi Ishibashi, Koji Takano, Namiko Nishida, Tomoko Shofuda, Ema Yoshioka, Daisuke Kanematsu, Mishie Tanino, Yoshinori Kodama, Masayuki Mano, Yonehiro Kanemura

**Affiliations:** Department of Neurosurgery, Asahikawa Medical University, Asahikawa, Japan; Department of Neurosurgery, Asahikawa Medical University, Asahikawa, Japan; Department of Neurosurgery, Osaka International Cancer Institute, Osaka, Japan; Department of Neurological Surgery, Wakayama Medical University School of Medicine, Wakayama, Japan; Department of Neurosurgery, Wakayama Rosai Hospital, Wakayama, Japan; Department of Neurosurgery, Asahikawa Medical University, Asahikawa, Japan; Department of Neurosurgery, Osaka General Medical Center, Osaka, Japan; Department of Neurosurgery, Asahikawa Medical University, Asahikawa, Japan; Department of Neurosurgery, Japanese Red Cross Kitami Hospital, Kitami, Japan; Department of Neurosurgery, Asahikawa Medical University, Asahikawa, Japan; Department of Neurosurgery, Wakayama Rosai Hospital, Wakayama, Japan; Department of Neurological Surgery, Wakayama Medical University School of Medicine, Wakayama, Japan; Department of Neurosurgery, Osaka University Graduate School of Medicine, Osaka, Japan; Department of Neurosurgery, NHO Osaka National Hospital, Osaka, Japan; Department of Neurosurgery, NHO Osaka National Hospital, Osaka, Japan; Department of Neurosurgery, Kansai Medical University, Hirakata, Japan; Department of Neurosurgery, Osaka Metropolitan University Graduate School of Medicine, Osaka, Japan; Department of Neurosurgery, Osaka International Cancer Institute, Osaka, Japan; Department of Neurosurgery, Osaka University Graduate School of Medicine, Osaka, Japan; Department of Neurosurgery, Yao Municipal Hospital, Yao, Japan; Department of Neurosurgery, Osaka City General Hospital, Osaka, Japan; Department of Neurosurgery, Osaka International Cancer Institute, Osaka, Japan; Department of Neurosurgery, Toyonaka Municipal Hospital, Toyonaka, Japan; Department of Neurosurgery, Tazuke Kofukai Foundation, Medical Research Institute, Kitano Hospital, Osaka, Japan; Department of Biomedical Research and Innovation, Institute for Clinical Research, NHO Osaka National Hospital, Osaka, Japan; Department of Biomedical Research and Innovation, Institute for Clinical Research, NHO Osaka National Hospital, Osaka, Japan; Department of Biomedical Research and Innovation, Institute for Clinical Research, NHO Osaka National Hospital, Osaka, Japan; Department of Diagnostic Pathology, Asahikawa Medical University Hospital, Asahikawa, Japan; Department of Neurosurgery, NHO Osaka National Hospital, Osaka, Japan; Department of Biomedical Research and Innovation, Institute for Clinical Research, NHO Osaka National Hospital, Osaka, Japan; Department of Diagnostic Pathology and Cytology, Osaka International Cancer Institute, Osaka, Japan; Department of Central Laboratory and Surgical Pathology, NHO Osaka National Hospital, Osaka, Japan; Department of Diagnostic Pathology and Cytology, Osaka International Cancer Institute, Osaka, Japan

**Keywords:** glioblastoma, magnetic resonance image, MGMT, MGMT promoter methylation, radiogenomics

## Abstract

**Background:**

The study aims to explore MRI phenotypes that predict glioblastoma’s (GBM) methylation status of the promoter region of *MGMT* gene (pMGMT) by qualitatively assessing contrast-enhanced T1-weighted intensity images.

**Methods:**

A total of 193 histologically and molecularly confirmed GBMs at the Kansai Network for Molecular Diagnosis of Central Nervous Tumors (KANSAI) were used as an exploratory cohort. From the Cancer Imaging Archive/Cancer Genome Atlas (TCGA) 93 patients were used as validation cohorts. “Thickened structure” was defined as the solid tumor component presenting circumferential extension or occupying >50% of the tumor volume. “Methylated contrast phenotype” was defined as indistinct enhancing circumferential border, heterogenous enhancement, or nodular enhancement. Inter-rater agreement was assessed, followed by an investigation of the relationship between radiological findings and pMGMT methylation status.

**Results:**

Fleiss’s Kappa coefficient for “Thickened structure” was 0.68 for the exploratory and 0.55 for the validation cohort, and for “Methylated contrast phenotype,” 0.30 and 0.39, respectively. The imaging feature, the presence of “Thickened structure” and absence of “Methylated contrast phenotype,” was significantly predictive of pMGMT unmethylation both for the exploratory (*p* = .015, odds ratio = 2.44) and for the validation cohort (*p* = .006, odds ratio = 7.83). The sensitivities and specificities of the imaging feature, the presence of “Thickened structure,” and the absence of “Methylated contrast phenotype” for predicting pMGMT unmethylation were 0.29 and 0.86 for the exploratory and 0.25 and 0.96 for the validation cohort.

**Conclusions:**

The present study showed that qualitative assessment of contrast-enhanced T1-weighted intensity images helps predict GBM’s pMGMT methylation status.

**Key Points**

- MRI features, the “Thickened structure” and the “Methylated contrast phenotype,” help predict GBM’s pMGMT methylation.
- The presence of “Thickened structure,” and absence of “Methylated contrast phenotype” has a specificity of 0.86–0.96 favoring pMGMT unmethylation.

**Importance of the Study**

Noninvasive prediction of glioblastoma’s (GBM) pMGMT methylation status is still a challenging research topic despite recent technological advancements in image analysis. Thus, this study explored a clinically feasible imaging biomarker that represents GBM’s pMGMT methylation status with external validation. Two qualitative imaging features, namely the “Thickened structure” and the “Methylated contrast phenotype,” were identified as valuable to this means. GBMs presenting the imaging feature, the presence of “Thickened structure” and absence of “Methylated contrast phenotype” exhibited a significantly high specificity, favoring pMGMT unmethylation in the exploratory and validation cohorts with a sensitivity and specificity of approximately 0.3 and 0.9. The easy clinical application of the proposed imaging features is expected to facilitate better preoperative GBM characterization.

Glioblastoma (GBM) is one of the most common malignant brain tumors with an abysmal prognosis despite multimodal treatments consisting of maximal safe resection followed by radiation and chemotherapy with temozolomide.^1^ Methylation of the gene’s promoter region encoding the O-6-methylguanine-DNA methyltransferase (MGMT) is a predictive and prognostic factor in GBM patients.^2–4^ Although the benefit of presurgical identification of MGMT promoter methylation (pMGMT-met) of GBMs is debatable, it may help clinicians and patients choose the most appropriate treatment strategy. For instance, aggressive surgical intervention could be proposed more strongly for MGMT promotor unmethylated (pMGMT-unmet) than for pMGMT-met GBM patients due to the expected minimal benefit from temozolomide.^5^ In contrast, an optimal balance between maximal resection and preservation of the patient’s quality of daily life could be considered for pMGMT-met GBM patients.

Great efforts have been made to meet this research community’s demand to develop methods for noninvasive prediction of the pMGMT methylation status in GBM by magnetic resonance image (MRI). Experimented techniques include qualitative image assessments,^6,7^ texture features,^8–10^ and deep learning architectures.^11–14^ Despite these efforts, the prediction of GBM’s pMGMT methylation status is still troubled by inconsistent research results,^7,15^ insufficient diagnostic performance, with sensitivity and specificity widely ranging from 55.6% to 93%, and 39.0% to 76.0%, respectively,^8,16^ and possible overfitting of the deep learning algorithm.^12,13^ Furthermore, texture feature analyses and deep learning architectures are still far from being incorporated into routine clinics, as they require sophisticated procedures, such as segmentation, manual intervention, in-house analytic pipeline, and lengthy processing time.^17^

We previously reported that the diagnostic performance of pMGMT methylation status was low, with a sensitivity of 67% and a specificity of 66% by structural MRI-based radiomics.^9^ However, tumors with irregular shapes pose a challenge in acquiring consistent radiomic data. Thus, qualitative evaluation of radiological images may still be a valuable approach to predict the molecular status of GBM preoperatively. Previous research harnessing deep learning algorithms reported that pMGMT-unmet gliomas tended to demonstrate thick enhancement with central necrosis. In contrast, heterogenous or nodular enhancement were features characteristic of pMGMT-met gliomas.^13^ These imaging features identified by the deep learning algorithm could potentially be applied to predict GBM’s pMGMT methylation status by qualitative evaluation of MRI. The current study attempted to discover qualitative MRI characteristics corresponding to GBM’s pMGMT methylation status and to test the hypothesis that conventional qualitative evaluation of MRI remains valid for predicting brain tumors’ molecular characteristics.

## Methods

### Patient Cohort

This study was performed per the principles of the Helsinki Declaration, and it was approved by the internal ethical review boards (Approval number 21040) and all collaborative institutes from the Kansai Molecular Diagnosis Network (KANSAI) for Central Nervous System Tumors, the list of which can be found in the acknowledgment section. Written informed consent was obtained from patients or their families for the prospectively recruited cohort. We also used the Cancer Imaging Archive (TCIA)/ Cancer Genome Atlas (TCGA) data set accessed on September 16, 2022,^18–20^ as an external validation cohort.

The Inclusion criteria for the present study were as follows: newly diagnosed GBM according to WHO Classification of Tumours Fifth Edition (WHO2021),^21^ available for both tumor’s pMGMT methylation status and preoperative gadolinium-enhanced T1-weighted images (T1WI-Gd). Cases without pMGMT methylation or IDH mutation status information, lack of postoperative images, or insufficient or atypical images were excluded from this study. There were 193 GBM, IDH-wildtype from 12 KANSAI institutions, with 97 being pMGMT-met and 96 being pMGMT-unmet GBM. The TCIA/ TCGA validation cohort comprised 93 GBM, IDH-wildtype with 49 pMGMT-met and 44 pMGMT-unmet GBM patients.

A supplementary cohort was established following the WHO Classification of Tumours, Revised Fourth Edition (WHO2016), specifically including IDH-mutant tumors.^22^ This cohort aims to cater to those interested in this subgroup. Detailed information is shown in Supplementary Tables 1 and 2. The KANSAI cohort consisted of 202 subjects, comprising 103 pMGMT-methylated and 99 pMGMT-unmethylated GBM cases. The TCIA/TCGA validation cohort consisted of 104 subjects, with 59 pMGMT-methylated and 45 pMGMT-unmethylated GBM.

### Genetic Analysis

Frozen or fresh tumor samples were obtained during surgery, and tumor genomic DNA was extracted from those tissues for genetic analysis. All Genetic analyses were performed at the Osaka National Hospital according to previously described procedures. Briefly, the methylation status of pMGMT was analyzed by quantitative methylation-specific PCR after bisulfite modification of genomic DNA, and a threshold of ≥1% was used for pMGMT methylation. The presence of hotspot mutations in *IDH1* (R132) and *IDH2* (R172) genes was analyzed by Sanger sequencing. A senior board-certified neuropathologist performed a central pathology review. Patient characteristics are described in Supplementary Tables 1 and 2. We obtained genetic information regarding *IDH* genes and pMGMT for the TCIA/ TCGA data set from the report by Cameron et al.^23^

### The Definition and Classification of Image Findings

**Figure 1.**
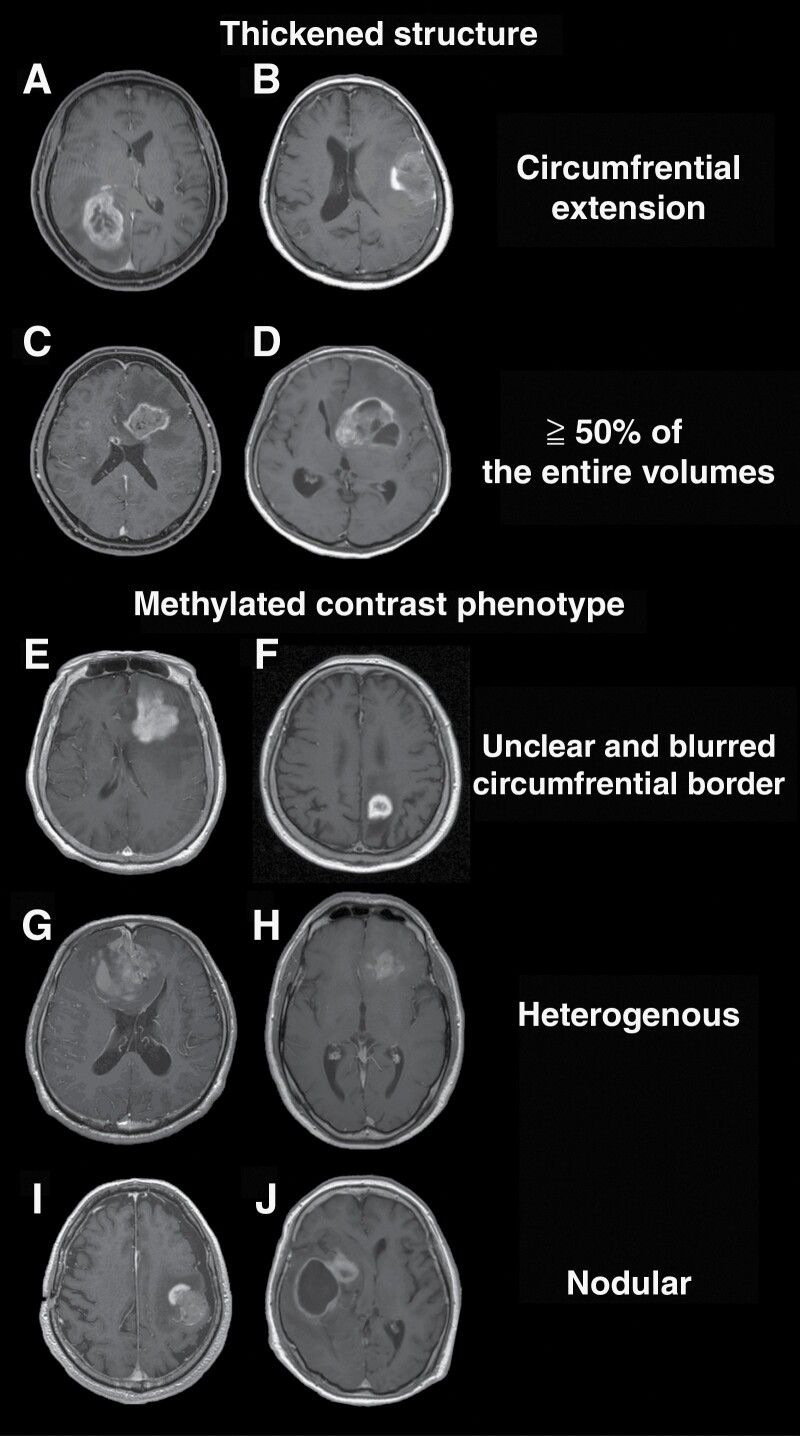
The “Thickened structure” was defined based on the presence of any of the 2 criteria: a solid circumferential component with central necrosis (**A**: KANSAI, Image 1D 35, pMGMT-met, **B**: KANSAI, Image 1D 15, pMGMT-met) or a solid component occupying more than 50% of the entire volume of the tumor (**C**: KANSAI, Image 1D 233, pMGMT-unmet, **D**: KANSAI, Image 1D 17, pMGMT-unmet). Methylated contrast phenotype” was defined by imaging findings with 1 of the 3 definitions: the entire enhancing circumferential border is unclear and blurred in relation to the surrounding brain (**E**: KANSAI, Image 1D 18, pMGMT-met, **F**: KANSAI, Image 1D 111, pMGMT-met), the enhancement is heterogenous (**G**: KANSAI, Image 1D 139, MGMT-met, **H**: KANSAI, Image 1D 218, pMGMT-met), or the enhancement is nodular (**I**: KANSAI, Image 1D 35, pMGMT-met, **J**: KANSAI, Image 1D 15, pMGMT-met).

**Figure 2.**
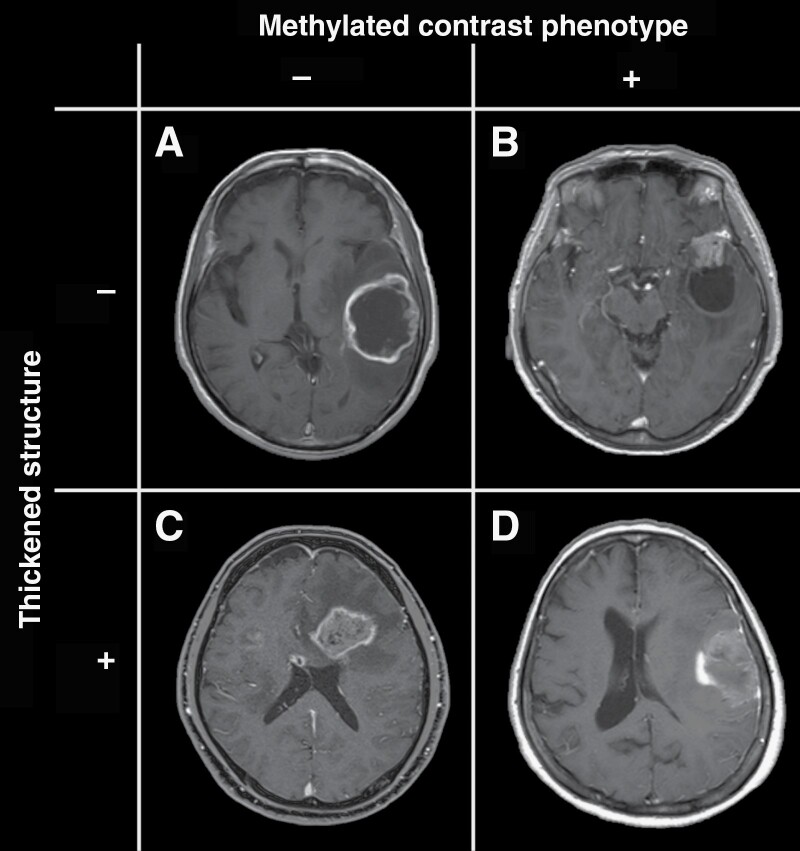
All cases were classified into 4 types: absence of both “Methylated contrast phenotype” and “Thickened structure” (**A**: KANSAI, Image 1D 210, pMGMT-unmet); presence of “Methylated contrast phenotype” and absence of “Thickened structure” (**B**: KANSAI, Image 1D 54, pMGMT-met); absence of “Methylated contrast phenotype” and presence of “Thickened structure” (**C**: KANSAI, Image 1D 233, pMGMT-unmet). Presence of both “Methylated contrast phenotype” and “Thickened structure” (**D**: KANSAI, Image 1D 35, pMGMT-met).

MRIs were independently evaluated by 3 board-certified neurosurgeons with 7, 8, and 12 years of experience blinded to tumors’ genetic information. The readers assessed the presence or absence of the following 2 T1WI-Gd characteristics; “Thickened structure” and “Methylated contrast phenotype” (Figure 1). These features were initially discovered as imaging characteristics useful for predicting glioma’s pMGMT methylation status by a deep learning algorithm in a previous study.^13^ The current study included the following definitions to clarify the description of imaging characteristics further. The presence of a “Thickened structure” was defined when the contrast-enhancing compartment of the tumor had either a circumferential extension (Figure 1A and B) or occupied more than 50% of the entire volume of the tumor (Figure 1C and D). A thin contrast-enhancing rim was defined as an absence of a “Thickened structure” (Figure 1A and B). The presence of a “Methylated contrast phenotype” was determined when the tumor showed *either one* of the following 3 features: 1. the entire enhancing circumferential border is unclear and blurred in relation to the surrounding structure (Figure 1E and F), 2. the enhancement is heterogenous (Figure 1G and H), 3. a nodular contrast enhancement is present (Figure 1I and J). Microvasculature-like minor sequential enhancements were not defined as “Methylated contrast phenotype” present (Figure 2C). The inter-rater consistencies among the 3 evaluators were evaluated using Fleiss’s Kappa coefficient. The final image findings were then determined by majority voting, and the cases were classified into 4 image types (Figure 2).

### Fleiss’s Kappa Coefficient and Statistical Analysis

Fleiss’s kappa coefficient was calculated using the “irr” package version 0.84.1 for R with default parameters (https://cran.r-project.org/web/packages/irr/irr.pdf). Fleiss’s kappa coefficient of 0.00 to 0.20 was considered as slight agreement, 0.21 to 0.40 as fair agreement, 0.41 to 0.60 as moderate agreement, and larger than 0.6 as substantial agreement.^24^ Statistical analysis was performed using Prism 9 for macOS (GraphPad Software, San Diego, CA, USA). The relationship between image characteristics and the pMGMT methylation status was investigated by the Fisher exact test or multiple logistic regression analysis. A *p* value of less than .05 was considered significant.

## Results

### Inter-rater Reliability of the “Thickened Structure” and the “Methylated Contrast Phenotype”

Inter-rater reliability of 3 evaluators assessing the “Thickened structure” was 0.68 for the KANSAI exploratory and 0.55 for the TCIA/ TCGA validation cohorts, suggesting substantial, and moderate agreements by Fleiss’s kappa coefficient, respectively. Regarding “Methylated contrast phenotype,” Fleiss’s kappa coefficients were 0.30 for the KANSAI and 0.39 for the TCIA/ TCGA cohorts, both of which implied fair agreement. The information regarding the agreement or disagreement between each reader can be referred to in Supplementary Tables 1 and 2.

### Correlation of Single Qualitative Image Phenotypes and pMGMT Methylation Both for the Exploratory and Validation Cohorts

**Figure 3.**
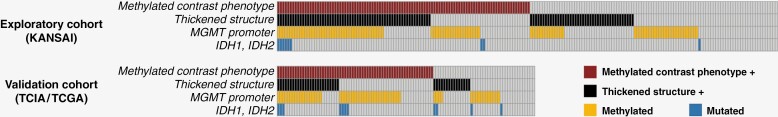
Overall study cohort. The study was conducted in 2 stages, an exploratory cohort study followed by a validation cohort study, to investigate the relationship between the imaging characteristics and the *pMGMT*-methylation status of histologically confirmed GBM. KANSAI, Kansai Molecular Diagnosis Network; TCIA/TCGA, Cancer Imaging Archive/ Cancer Genome Atlas.

Figure 3 shows the presence or absence of qualitative image phenotypes and molecular status of the 2 cohorts. A multiple logistic analysis for the exploratory cohort (KANSAI) following the WHO2021 criteria revealed that pMGMT methylation was predicted by the following equation:

MGMT (unmethy:0, methy:1) approximately −0.39 + 0.01 x “Thickened structure (absent:0, present:1)” + 0.82 x “Methylation contrast phenotype (absent:0, present:1)”

**Figure 4.**
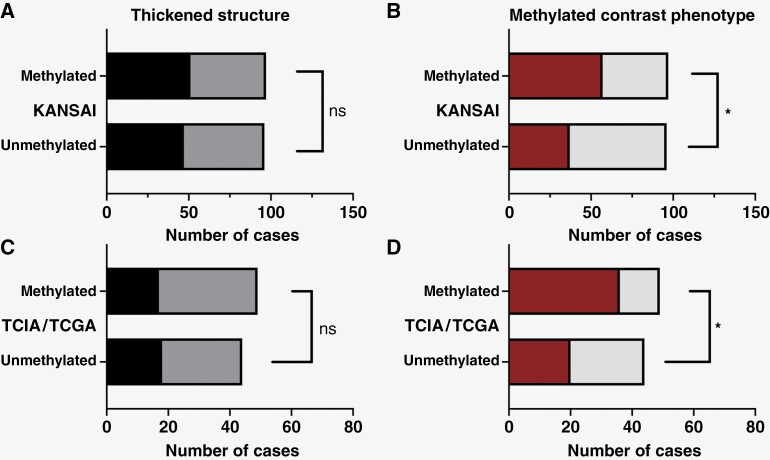
Exploration of the relationship between the imaging characteristics and the pMGMT-methylation status of GBMs. “Thickened structure” was not significantly associated with pMGMT-met GBMs (KANSAI cohort **(A)**, *p* = 0.98 and TCIA/ TCGA cohort **(C)**, *p* = 0.44). “Methylated contrast phenotype” was significantly associated with pMGMT-met of GBMs (KANSAI cohort **(B)**, *p* = 0.006 and TCIA/ TCGA cohort **(D)**, *p* = 0.006)

Although the “Thickened structure” did not significantly contribute to the model construction (*p* = .98, Figure 4A), “Methylation contrast phenotype” was considered significant (*p* = .006, Figure 4B). This trend was consistently observed in the validation cohort as well (TCIA/ TCGA; Figure 4C and D).

### Correlation of the Combined Qualitative Image Phenotypes and pMGMT Methylation Both for the Exploratory and Validation Cohorts

**Figure 5.**
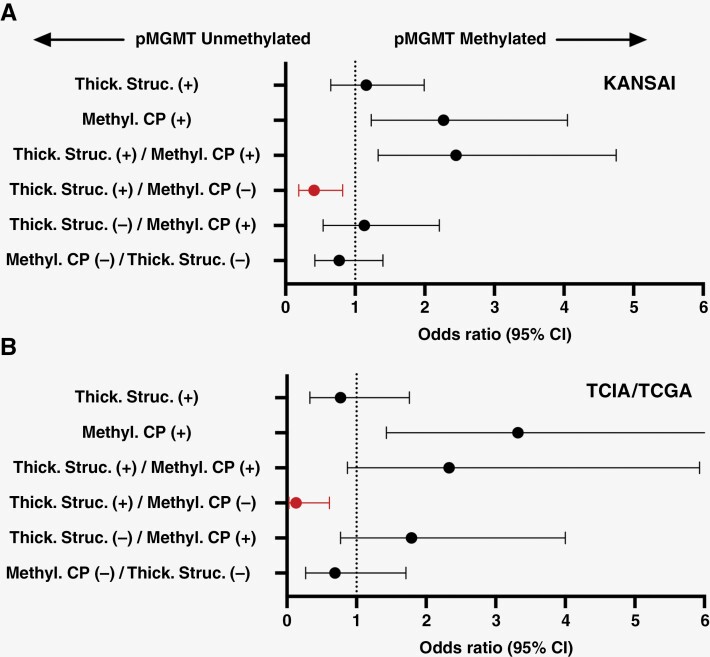
The odds ratios (OR) of the imaging characteristics for predicting GBM’s pMGMT methylation status are presented. The OR of the “Methylated contrast phenotype” (Methyl. CP) was significantly higher than 1.0 in both the KANSAI exploratory and TCIA/ TCGA validation cohorts. The OR of the presence of “Thickened structure” (Thick. Struc.) and the absence of “Methylated contrast phenotype” was significantly lower than 1.0 both in the KANSAI exploratory and TCIA/ TCGA validation cohort.

Fisher exact test revealed that the presence of both the “Thickened structure” and “Methylated contrast phenotype” was significantly predictive of pMGMT-met GBM for the KANSAI exploratory (Figure 5A and Supplementary Figure 1A; *p* = .007, odds ratio = 2.50, 95% confidence interval [CI] = 1.33–4.76). However, there was no significant difference in TCIA/ TCGA validation cohorts (Figure 5B and Supplementary Figure 2A; *p* = .14, odds ratio = 2.32, 95% CI = 0.87–5.93). The sensitivities and specificities of the “Thickened structure” and “Methylated contrast phenotype” double positive for correctly predicting pMGMT-met GBM were 0.66 and 0.80 for the KANSAI exploratory and 0.31 and 0.84 for the TCIA/ TCGA validation cohort (Table 1).

**Table 1.** Odds Ratio and Diagnostic Performance of the “Thickened Structure” and “Methylated Contrast Phenotype” for pMGMT Methylation Status in GBM. † Indicates *p* < 0.05.

		Positive number	*p* Value	pMGMT-Met GBM prediction				pMGMT-Unmet GBM prediction			
				Odds ratio	95% CI	Sensitivity	Specificity	Odds ratio	95% CI	Sensitivity	Specificity
KANSAI cohort (exploratory) Total 193 subjects	Thick. Struc. (+)	98	0.67	1.16	0.65–1.99	52.0%	51.0%	0.87	0.50–1.55	49.0%	47.4%
	Methyl. CP (+)	94	0.01^†^	2.27	1.23–4.05	60.6%	61.5%	0.44	0.25–0.80	38.5%	41.2%
	Thick. Struc.(+)/ Methyl. CP (+)	56	0.01^†^	2.45	1.33–4.75	66.1%	80.2%	0.40	0.21–0.75	19.8%	61.9%
	Thick. Struc.(+)/ Methyl. CP (-)	42	0.01^†^	0.41	0.19–0.82	33.3%	70.8%	2.44	1.21–5.15	29.2%	85.6%
	Thick. Struc.(-)/ Methyl. CP (+)	38	0.86	1.13	0.54–2.21	52.6%	81.3%	0.89	0.45–1.85	18.8%	79.4%
	Thick. Struc.(-)/ Methyl. CP (-)	57	0.43	0.77	0.42–1.40	45.6%	67.7%	1.30	0.72–2.41	32.3%	73.2%
TCIA/ TCGA cohort (validation) Total 93 subjects	Thick. Struc. (+)	35	0.67	0.77	0.33–1.76	34.7%	59.1%	1.30	0.57–3.03	40.9%	65.3%
	Methyl. CP (+)	56	0.01^†^	3.32	1.43–7.63	73.5%	54.6%	0.30	0.13–0.70	45.5%	26.5%
	Thick. Struc.(+)/ Methyl. CP (+)	22	0.14	2.33	0.87 – 5.93	30.6%	84.1%	0.43	0.17–1.15	15.9%	69.4%
	Thick. Struc.(+)/ Methyl. CP (-)	13	0.01^†^	0.13	0.03–0.61	4.1%	75.0%	7.83	1.63–36.56	25.0%	95.9%
	Thick. Struc.(-)/ Methyl. CP (+)	34	0.20	1.79	0.77–4.00	42.9%	70.5%	0.56	0.25–1.30	29.6%	57.1%
	Thick. Struc.(-)/ Methyl. CP (-)	24	0.48	0.69	0.27–1.71	22.5%	70.5%	1.45	0.58–3.77	29.6%	77.6%

Further investigating the correlation of the 2 qualitative image features to GBM’s pMGMT methylation status, the presence of the “Thickened structure” and absence of Methylated contrast phenotype” significantly correlated with GBM’s pMGMT unmethylation both for the KANSAI exploratory cohort (Figure 5A red colored datapoint and Supplementary Figure 1B, *p* = .015, odds ratio = 2.44, 95% CI = 1.21–5.15) and the TCIA/ TCGA validation cohort (Figure 5B red colored datapoint and Supplementary Figure 2B, *p* = .006, odds ratio = 7.83, 95% CI = 1.63–36.56). The sensitivities and specificities for correctly predicting pMGMT-unmet GBM referring to the presence of the “Thickened structure” and absence of Methylated contrast phenotype” were 0.29 and 0.86 for the KANSAI exploratory and 0.25 and 0.96 for the TCIA/ TCGA validation cohort (Table 1) based on the WHO2021 criteria. On the other hand, different combinations of the qualitative image features did not correlate with GBM’s pMGMT methylation status (Supplementary Figures 3 and 4). The reestablished cohort based on the WHO2016 criteria also confirmed these tendencies (Supplementary Figures 3 and 4; Supplementary Table 3).

## Discussion

The pMGMT methylation status is known to be one of the most important prognostic and predictive factors of GBM,^25^ and preoperative prediction of the pMGMT methylation status could benefit patient care, providing the possibility of identifying poor prognostic patients and offering them more aggressive or experimental treatments. Although radiomics and deep learning are now under extensive investigation, the diagnostic performance of predicting pMGMT methylation ranges widely in sensitivity and specificity from 55.6% to 93% and from 39.0% to 76.0%, respectively.^8–10,12,13,16^ There could also be issues related to the data used for training, such as an unbalanced training data set and overestimation of diagnostic performance.^13,17^ Moreover, applying these relatively complicated procedures to daily clinical practice is also a significant challenge.

A qualitative visual assessment of radiological images does not require any complicated analytical pipeline, which can be readily incorporated into clinical practice. Previous studies reported that ill-defined tumor margin was seen more frequently in high-grade gliomas with pMGMT methylation^7^ and ring enhancement in pMGMT-unmet GBM.^6,8^ However, incongruent studies showed no correlation between these findings and the pMGMT methylation status of the tumors.^7,15^ The present study demonstrated that the newly defined “Methylated contrast phenotype” highly correlated with pMGMT-met GBM in many patients in 2 independent cohorts. Moreover, this is the first report to predict GBM’s pMGMT methylation status by qualitative visual assessment based on the WHO Classification of Tumours Fifth Edition (WHO2021).^21^

Furthermore, we revealed high specificity for predicting pMGMT-unmet GBM by combining the 2 imaging features: the presence of “Thickened structure” and the absence of “Methylated contrast phenotype.” The specificities were 85.6% in the exploratory cohort and 95.9% in the validation cohort, better than those using radiomics and deep learning.^8–10,16^ Similar to the T2-FLAIR mismatch sign for detecting IDH-mutant astrocytoma, which is a clinically relevant imaging phenotype with low sensitivity but high specificity,^26–29^ the current study suggested that a particular GBM population that exhibits specific imaging characteristics can be predicted to be pMGMT unmethylated with high specificity. These findings indicate the potential for predicting the effectiveness of chemotherapeutic agents in GBM patients before surgery,^30,31^ providing valuable information in the decision-making process regarding intraoperative implantation of BCNU wafers.

It is important to note that the inter-rater agreements for both qualitative assessments were deemed more than “fair,” underscoring their validity as functional imaging characteristics. However, the proposed imaging characteristics in the present study, aimed at predicting GBM’s pMGMT methylation status, fall short of being satisfactory for preoperative assessment in bedside clinics. While additional research incorporating other imaging characteristics retrievable from T2-weighted images, fluid-attenuated inversion recovery (FLAIR), and apparent diffusion coefficient may enhance diagnostic accuracy,^7,13^ we anticipate challenges in this research domain. A recently published article, which focuses on MRI-based prediction of GBM’s MGMT methylation status powered by deep learning models, highlights the profound difficulty of achieving this task.^32^ In contrast to previous studies, the current research takes a different approach by attempting to identify a subgroup of GBM where the MGMT methylation status can be readily discerned rather than solely focusing on the overall prediction accuracy of the diagnostic model. Furthermore, it delves into qualitative visual assessment, aiming for an approach easily applicable in daily clinical practice without the need for complicated analytical pipelines.

Several limitations of the present study must be addressed. First, while the present study is the first to evaluate qualitative imaging features to predict GBM’s pMGMT methylation status in a 2-staged fashion with a larger sample size than previous similar studies,^6–8,15^ the retrospective design requires a prospective study with a larger sample size to validate our findings further. Second, the inter-rater agreement was not excellent, especially in the “Methylated contrast phenotype,” with Fleiss’s kappa coefficient being 0.30 for the KANSAI cohort and 0.39 for the TCIA/ TCGA cohort, which might limit the generalizability of the proposed imaging feature. Furthermore, although this study was based on 2 independent cohorts, the qualitative representation of images may differ among different cohorts. Confounding factors that influence image characteristics, such as MRI vendors and inconsistent MRI acquisition parameters, are another issue that may negatively affect inter-rater agreement. Third, the methods used for detecting pMGMT methylation must also be addressed, as different methods and various cutoffs are available to identify GBM’s pMGMT methylation status.^33^ This issue could be problematic in generalizing the current finding and could affect the “ground truth” for establishing any diagnostic model. Fourth, the current research used sensitivity and specificity as the endpoint of the analysis due to the binary assignment of each image finding. Many deep learning-based research studies report the area under the curve as its primary outcome,^11,14,32,34^ which hampers direct diagnostic accuracy comparison with the current research. Last, the present study did not assess the intra-rater consistency over time, which could significantly affect diagnostic reproducibility.

In conclusion, the present study showed that qualitative assessment of contrast-enhanced T1-weighted intensity images is useful to predict GBM’s pMGMT methylation status, and the proposed “Thickened structure” and “Methylated contrast phenotype” are valuable image biomarkers to better understand the GBM’s pMGMT methylation status in a preoperative setting.

## Supplementary Material

Supplementary material is available online at *Neuro-Oncology* (https://academic.oup.com/neuro-oncology).

vdae016_suppl_Supplementary_Tables_S1

vdae016_suppl_Supplementary_Figures_S1-S4

vdae016_suppl_Supplementary_Tables_S3

vdae016_suppl_Supplementary_Tables_S2

## Data Availability

Anonymized clinical and demographic data of the KANSAI cohort, radiological data, and other relevant data are provided in Supplementary Table 1. The senior authors and the Kansai Network for Molecular Diagnosis of Central Nervous Tumors will review requestable requests for additional data to determine whether they can be fulfilled following the privacy restrictions of each participating institution. Requests for additional materials related to this work should be directed to M.K.
