# Supplementary material for: Prediction of MGMT promotor methylation status in glioblastoma by contrast-enhanced T1-weighted intensity image
Source: Neurooncol Adv. 2024 Feb 1;6(1):vdae016. doi: 10.1093/noajnl/vdae016 (PMC10896622; doi:10.1093/noajnl/vdae016)
Supplement: vdae016_suppl_Supplementary_Figures_S1-S4 [file vdae016_suppl_supplementary_figures_s1-s4.docx]

**Supplementary figures: Prediction of MGMT promotor methylation status in glioblastoma by contrast-enhanced T1-weighted intensity image**


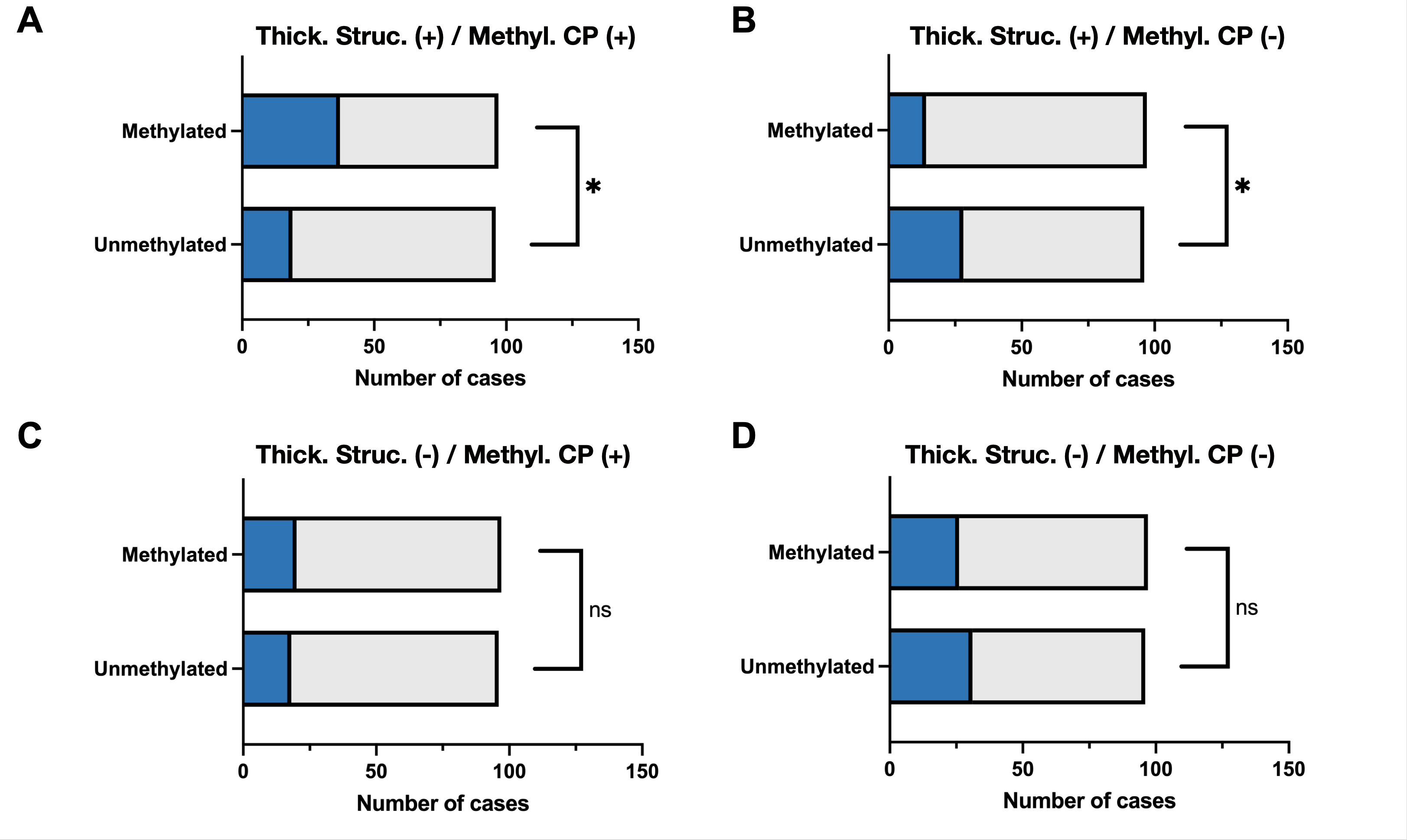


**Figure S1.** The correlations between pMGMT methylation status and the combined qualitative image phenotypes, “Thickened structure” (Thick. Struc.) and “Methylation contrast phenotype” (Methyl. CP) for the KANSAI cohort are shown. “Thickened structure” and “Methylated contrast phenotype” double positive significantly correlated with pMGMT methylation (**A**, *p* = 0.007). The presence of “Thickened structure” and the absence of “Methylated contrast phenotype” were significantly associated with pMGMT unmethylation (**B**, *p* = 0.015). The other combinations of qualitative image phenotypes did not correlate with GBM’s pMGMT methylation status (**C**, *p* = 0.86, **D**, *p* = 0.43).
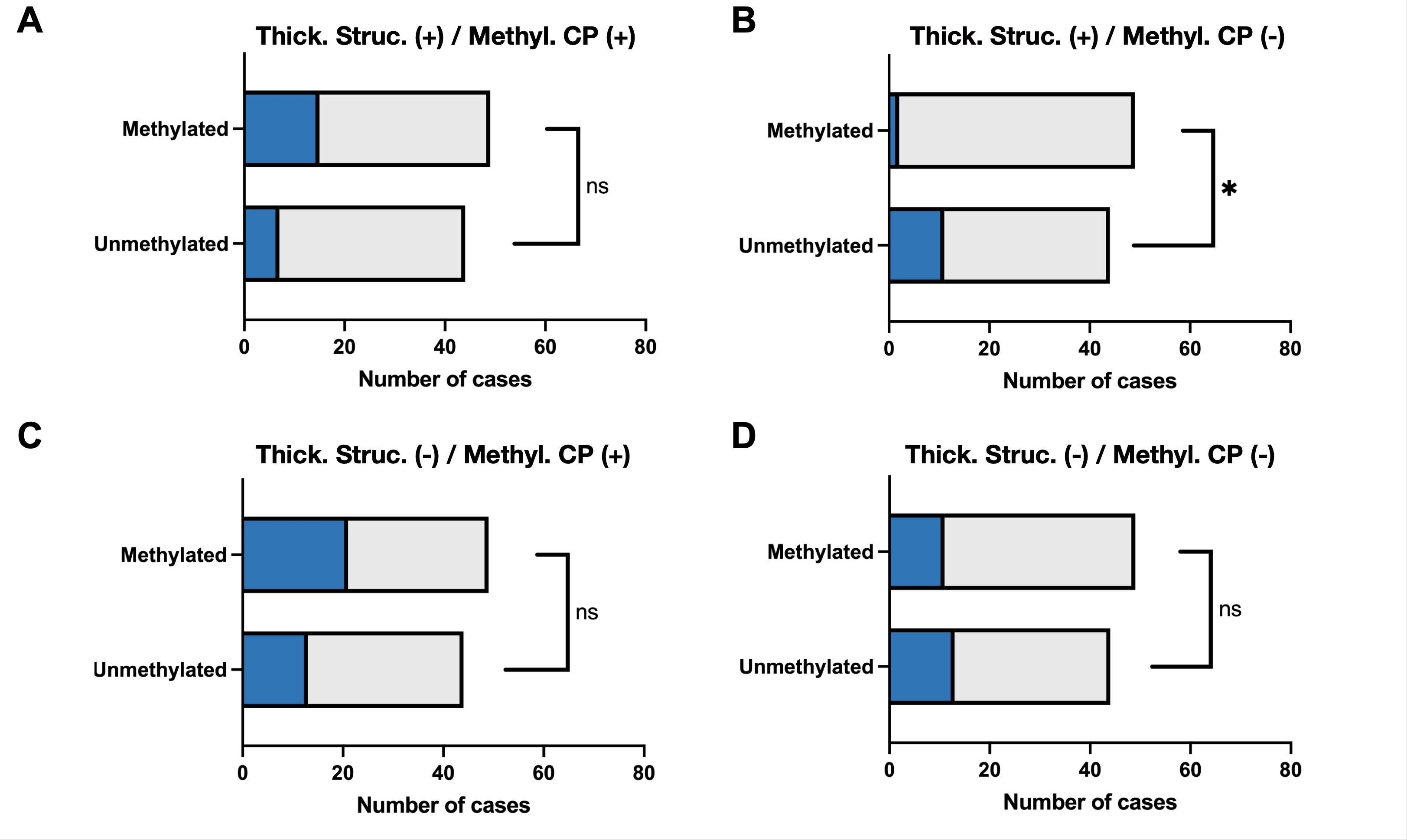


**Figure S2.** The correlation between pMGMT methylation status and the combined qualitative image phenotypes, “Thickened structure” (Thick. Struc.) and “Methylation contrast phenotype” (Methyl. CP) for the TCIA / TCGA cohort are shown. The presence of “Thickened structure” and the absence of “Methylated contrast phenotype” were significantly associated with pMGMT unmethylation (**B**, *p* = 0.006). The other combinations of qualitative image phenotypes did not correlate with GBM’s pMGMT methylation status (**A**, *p* = 0.14, **C**, *p* = 0.20, **D**, *p* = 0.48).


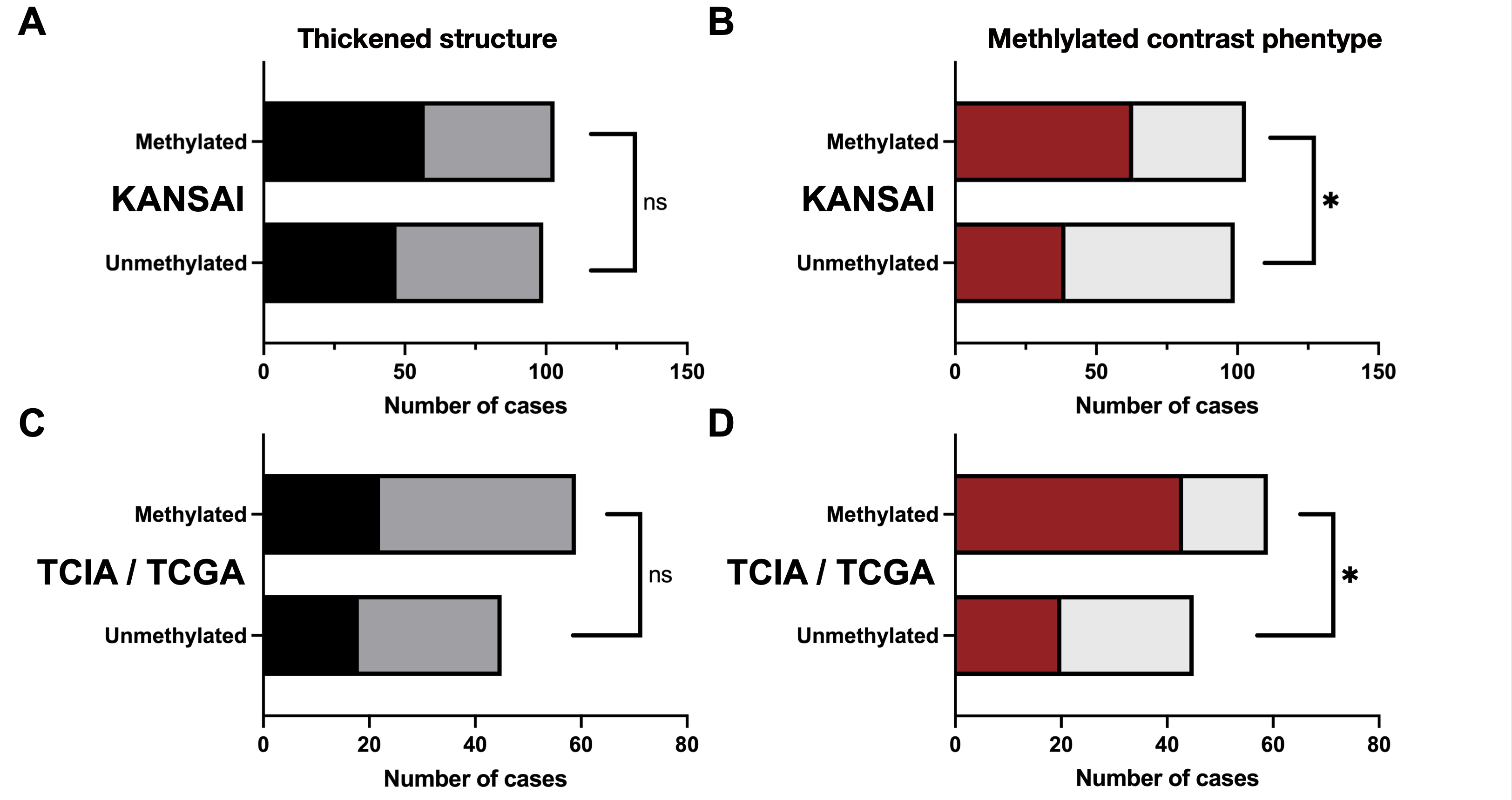


**Figure S3.** Exploration of the relationship between the imaging characteristics and the pMGMT-methylation status of GBMs in the cohort according to the WHO Classification of Tumours, Revised 4th Edition (WHO2016), including IDH-mutant tumors. “Thickened structure” was not significantly associated with pMGMT-met GBMs (KANSAI cohort **(A)**, *p* = 0.58 and TCIA / TCGA cohort **(C)**, *p* = 0.70). “Methylated contrast phenotype” was significantly associated with pMGMT-met of GBMs (KANSAI cohort **(B)**, *p* = 0.004 and TCIA / TCGA cohort **(D)**, *p* = 0.004)


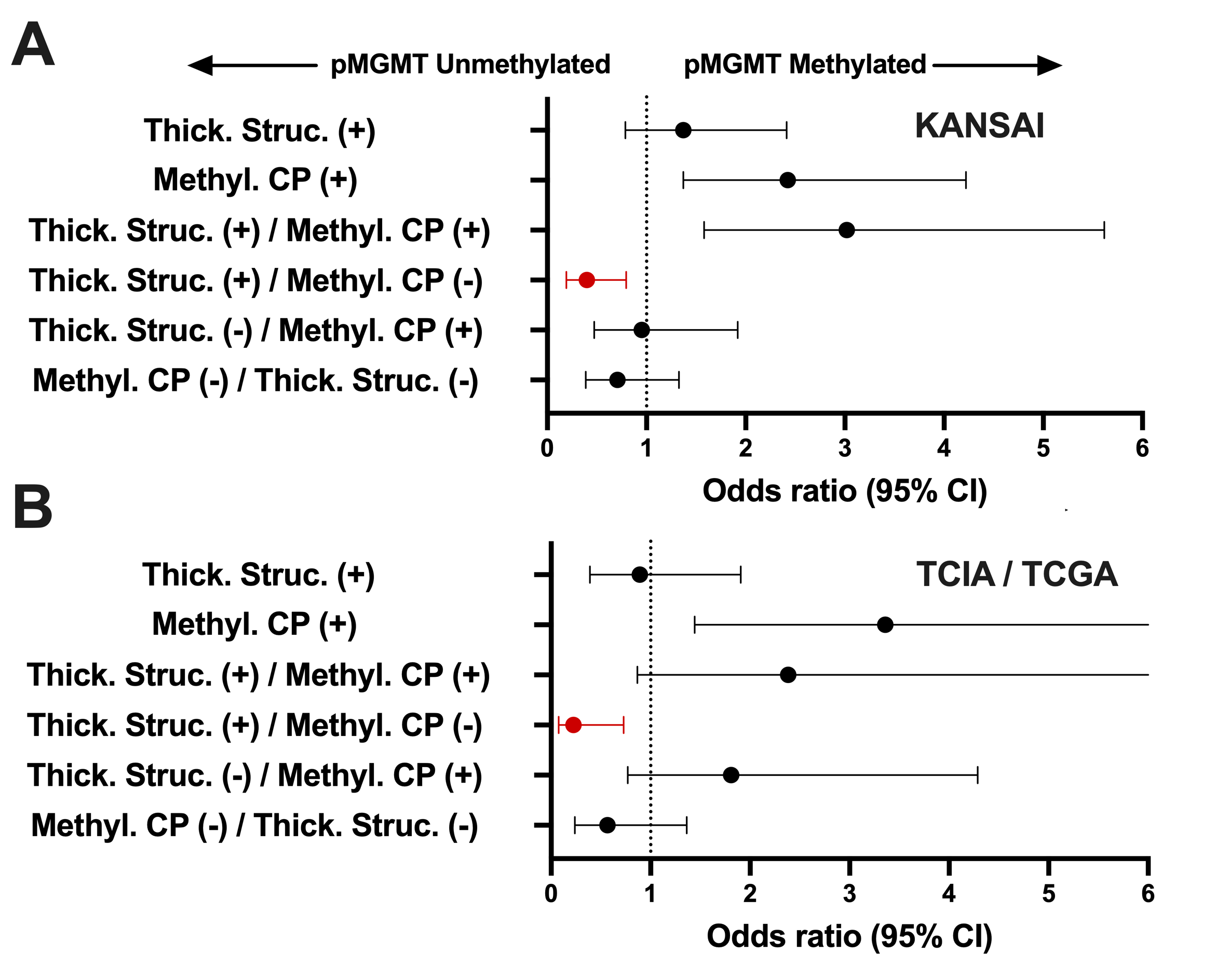


**Figure S4.** The odds ratios (OR) of the imaging characteristics for predicting GBM’s pMGMT methylation status in the cohort according to the WHO Classification of Tumours, Revised 4th Edition (WHO2016), including IDH-mutant tumors. The OR of the “Methylated contrast phenotype” (Methyl. CP) was significantly higher than 1.0 in both the KANSAI exploratory and TCIA / TCGA validation cohorts. The OR of the presence of “Thickened structure” (Thick. Struc.) and the absence of “Methylated contrast phenotype” was significantly lower than 1.0 both in the KANSAI exploratory and TCIA / TCGA validation cohort.
